# *CYP2C8* and *CYP2C9* polymorphisms in relation to tumour characteristics and early breast cancer related events among 652 breast cancer patients

**DOI:** 10.1038/sj.bjc.6605428

**Published:** 2009-11-24

**Authors:** H Jernström, E Bågeman, C Rose, P-E Jönsson, C Ingvar

**Affiliations:** 1 Department of Oncology, Clinical Sciences, Lund University, Barngatan 2B, Lund SE-221 85, Sweden; 2 Faculty of Health and Society, Malmö University, Malmö, Sweden; 3 Department of Oncology, Lund University Hospital, Lund, Lund SE-221 85, Sweden; 4 Department of Surgery, Helsingborg Hospital, Helsingborg SE-251 87, Sweden; 5 Department of Clinical Sciences UMAS, Malmö, Sweden; 6 Department of Surgery, Lund University Hospital, Lund SE-221 85, Sweden

**Keywords:** CYP2C8, CYP2C9, polymorphism, disease-free survival, tamoxifen

## Abstract

**Background:**

*CYP2C8/9* polymorphisms may influence breast cancer-free survival after diagnosis due to their role in the metabolism of tamoxifen, paclitaxel, and other chemotherapy. cytochrome P450 (CYP)2C8/9 metabolise arachidonic acid to epoxyeicosatrienoic acids, which enhance migration and invasion *in vitro* and promote angiogenesis *in vivo.* We aimed to investigate the frequency of *CYP2C8/9* polymorphism*s* in relation to breast tumour characteristics and disease-free survival.

**Methods:**

A prospective series of 652 breast cancer patients from southern Sweden was genotyped for CYP2C8*3, CYP2C8*4, CYP2C9*2, and CYP2C9*3. Blood samples and questionnaires were obtained pre- and postoperatively. Clinical information and tumour characteristics were obtained from patients' charts and pathology reports.

**Results:**

Frequencies of *CYP2C8/9* polymorphisms were similar to healthy European populations. Significantly less node involvement (*P*=0.002) and fewer PR+ tumours (*P*=0.012) were associated with CYP2C8*4. Median follow-up was 25 months and 52 breast cancer-related events were reported. In a multivariate model, CYP2C8/9*3/*1*/*2/*1 was the only factor associated with increased risk for early events in 297 tamoxifen-treated, ER-positive patients, adjusted HR 2.54 (95%CI 1.11–5.79). The effect appeared to be driven by CYP2C8*3, adjusted HR 8.56 (95%CI 1.53–51.1).

**Conclusion:**

Polymorphic variants of *CYP2C8/9* may influence breast tumour characteristics and disease-free survival in tamoxifen-treated patients.

In Sweden, approximately 7000 women are diagnosed with breast cancer annually and 1500 die of their disease. Up to 25% of breast cancer patients considered to be at low risk for recurrence, that is, stage-I or II without lymph node involvement, recur within 5 years (Malmström *et al*, 2001, 2003). Approximately 15% of all breast cancer patients in Sweden die from their disease within 5 years and 30% die within 10 years of diagnosis. Adjuvant therapies such as radiation, tamoxifen, aromatase inhibitors (AIs), and chemotherapy improve the prognosis, but also confer a risk of adverse side effects (Early breast cancer trialists' collaborative group, 2005; Forbes *et al*, 2008). Moreover, many patients receive adjuvant therapy without any impact on survival, as most are already cured by surgery alone or the adjuvant therapy chosen does not work as intended. Markers, which would help to better tailor adjuvant therapy to each patient, are urgently needed.

Several genetic polymorphisms in genes such as cytochrome-P450 (CYP)*2C8* and *CYP2C9*, may influence survival after cancer diagnosis due to their role in the metabolism of various breast cancer drugs, including tamoxifen and chemotherapy (Jin *et al*, 2005). CYP2C8 and CYP2C9 are polymorphic enzymes. CYP2C8*3 and CYP2C9*2 are the major variant alleles in Caucasian populations (Yasar *et al*, 2002). Approximately 96% of subjects with the CYP2C8*3 allele also carried a CYP2C9*2 and 85% of subjects who had the CYP2C9*2 variant also carried a CYP2C8*3. CYP2C8*3 is defective in the metabolism of two important CYP2C8 substrates: the anticancer drug paclitaxel (Bahadur *et al*, 2002) and the physiologically important compound arachidonic acid (AA) (Dai *et al*, 2001). In addition, variants CYP2C8*4 and CYP2C9*2 and CYP2C9*3 also have a lower metabolic activity than the wild-type variants (Bahadur *et al*, 2002; Griskevicius *et al*, 2003; King *et al*, 2004; Sandberg *et al*, 2004).

Arachidonic acid is metabolised via three major pathways: the cyclooxygenase pathway, which produces prostaglandins; the lipoxygenase pathway, and finally the CYP epoxygenase pathway (Belton and Fitzgerald, 2003).

CYP2C8 and 2C9 are CYP epoxygenases, which metabolise AA to epoxyeicosatrienoic acids (EETs) (Zeldin *et al*, 1995; Michaelis *et al*, 2005), with the most abundant product being 14,15-EET, which promotes angiogenesis *in vivo* (Medhora *et al*, 2003). *In vitro* studies have shown that overexpression of CYP2C9 elicits angiogenesis via activation of the epidermal growth factor receptor (EGFR) (Michaelis *et al*, 2003). Jiang *et al* (2007) showed that CYP epoxygenase overexpression enhanced tumour metastasis of MDA-MB-231 human breast carcinoma cells to the lungs of athymic BALB/C mice. Moreover, CYP epoxygenase overexpression or EET treatment markedly enhanced the migration, invasion, and prometastatic gene expression profiles in a variety of cancer cell lines *in vitro* (Jiang *et al*, 2007). Functional polymorphisms of *CYP2C8* and *CYP2C9* may thus be of importance for breast cancer risk, tumour characteristics, and treatment response.

Our study has four interrelated aims: first, to investigate the frequency of the polymorphic variants CYP2C8*3, CYP2C8*4, CYP2C9*2, and CYP2C9*3 in a series of breast cancer patients; second, to construct *CYP2C8/9* haplotypes; third to determine whether these genetic variants were associated with breast cancer characteristics of prognostic importance; and fourth, to investigate whether any of these genetic variants were associated with early breast cancer-related events.

## Materials and methods

Women assessed preoperatively at the Lund University Hospital and the Helsingborg Hospital, Sweden, for a first breast cancer were invited to take part in an ongoing study regarding genetic and non-genetic factors that could be associated with breast cancer prognosis and treatment response. Patients were included between October 2002 and October 2007 in Lund, and between April 2006 and October 2007 in Helsingborg. Helsingborg is located approximately 50 km north of Lund. There are nine hospitals in the South Swedish Health Care Region performing breast cancer surgery. The Lund University Hospital catchment area serves almost 300 000 inhabitants and the Helsingborg Hospital serves another 250 000 inhabitants. Breast cancer patients are not referred to other hospitals for surgery. We, therefore, consider this study population-based.

Women were invited to participate regardless of ethnic background, age, and tumour stage. Patients who had been diagnosed recently and treated for another type of cancer within the past 10 years were not eligible to participate. The study was approved by the Ethics Committee of Lund University. Written informed consents were collected during the preoperative visit at the Department of Surgery at the Lund University Hospital. At the same visit, the research nurse collected blood samples (EDTA–plasma and serum) and recorded the time and date when the blood samples were drawn. The blood was centrifuged and separated. Serum, EDTA–plasma, and blood cells were stored at −70°C. All samples were labelled with serial codes to enable blinded analyses.

Body weight, height, waist and hip circumferences, and breast volumes were measured at the preoperative visit. All patients filled out a preoperative questionnaire including questions on birth date, coffee consumption, smoking, alcohol intake, use of exogenous hormones and concomitant medications, reproductive history, and family history of cancer. No question enquired about ethnicity. However, the vast majority of women included were ethnic Swedes.

Information including type of surgery, sentinel node biopsy, and axillary node dissection was obtained from each patient's chart. Tumour size, histological type and grade, axillary node involvement, signs of distant metastases, ER, and progesterone receptor (PR) status were obtained from each patient's pathology report.

Histological grade was evaluated according to the procedure of Elston and Ellis (1991). The grading procedure consisted of judgement of tubule formation, nuclear pleomorphism, and mitotic count. The number of mitoses was counted in 10 high-power fields, and the results were adjusted to the area of the microscopic field. Each of the three morphological features, tubules, nuclear pleomorphism, and number of mitoses, was given a score of 1 to 3 points. The overall histological grade was obtained by adding the score of each characteristic, resulting in a possible total score of 3 to 9 points. The histological grade allocation was as follows: grade-1, 3 to 5 points; grade-2, 6 to 7 points; and grade-3, 8 to 9 points.

Oestrogen receptor and PR status was determined by immunohistochemistry using the Dako LSAB kit system (Dako, Glostrup, Denmark) and antibodies M7047 (ER) and M3569 (PR) (Dako) in Lund. In Helsingborg, ER and PR status was determined by immunohistochemistry using the Ventana ultra view kit (760-500) (Ventana, Illkirch, France) and antibodies 790-4324 (ER) (Ventana) and NCL-L-PGR-312 (PR) (NovoCastra, NewCastle, UK). Tumours with more than 10% positive nuclear staining were considered ER+ or PR+. Receptor-negative tumours had a positive nuclear staining of 10% or less. All tumours were analysed at the Department of Pathology of Lund University Hospital or Helsingborg Hospital. HER-2/neu status was routinely analysed as of November 2005.

According to data obtained from the Regional Tumor Registry, on June 25, 2008, a total of 6765 primary female breast cancers were registered between October 1, 2002, and October 31, 2007, in the South Swedish Health Care Region. During the same time period, 893 breast tumours were registered in Lund, of which 866 were primary and received surgery. Five hundred and seventeen of them (60%) were included in our study. Between April 1, 2006, and October 31, 2007, a total of 330 breast cancers were registered at Helsingborg, of which 298 received surgery, and 136 (46%) of these were included in our study.

### Genetic analyses

Genomic DNA was extracted from 300 *μ*l of peripheral blood using the Wizard genomic DNA purification kit (Promega, Madison, WI, USA).

CYP2C8*3 consists of two polymorphisms (G416A) (rs11572080) and (A1196G) (rs10509681). CYP2C8*3 (G416A) (rs11572080) was amplified using PCR primers Fw: 5′-NNCCACCCTTGGTTTTTCTCAACTC-3′ and Re: 5′-BIOTIN-CCTCACAACCTTGCGGAATTT-3′ (Biomers, Ulm, Germany), which yield a 105-bp nucleotide sequence. PCR was performed in 25-*μ*l reactions using 25 ng DNA, 0.2 *μ*M of each primer, 0.2 mM of each deoxynucleotide (Amersham Biosciences, Buckinghamshire, UK), 1.5 mM MgCl_2_ (Applied Biosystems, Foster City, CA, USA), 1 × PCR Gold Buffer (Applied Biosystems), and 0.5 U of AmpliTaq Gold (Applied Biosystems).

CYP2C8*3 (A1196G) (rs10509681) was amplified using PCR primers Fw: 5′-BIOTIN-TTTGTTACTTCCAGGGCACA-3′ and Re: 5′-NNAAAGTGGCCAGGGTCAAAG-3′ (Biomers), which yield a 101-bp nucleotide sequence. PCR was performed in 25-*μ*l reactions using 25 ng DNA, 0.2 *μ*M of each primer, 0.2 mM of each deoxynucleotide (Amersham Biosciences), 1.5 mM MgCl_2_ (Applied Biosystems), 1 × PCR Gold Buffer (Applied Biosystems), and 0.5 U of AmpliTaq Gold (Applied Biosystems). Both SNPs were run for 298 samples with 100% concordance. We, therefore, genotyped only (rs11572080) for the remaining 354 samples.

CYP2C8*4 (rs1058930) was amplified using PCR primers Fw: 5′-NNGTTTCCCAGGAACTCACAACAAAG-3′ and Re: 5′-BIOTIN-AAGCATTACTGGCCTGATCATTT-3′ (Biomers), which yield a 255-bp nucleotide sequence. PCR was performed in 25-*μ*l reactions using 25 ng DNA, 0.17 *μ*M of each primer, 0.5 mM of each deoxynucleotide (Amersham Biosciences), 2.0 mM MgCl_2_ (Applied Biosystems), 1 × PCR Gold Buffer (Applied Biosystems), and 0.5 U of AmpliTaq Gold (Applied Biosystems).

CYP2C9*2 (rs1799853) was amplified using PCR primers Fw: 5′-NNGTATTTTGGCCTGAAACCCATA-3′ and Re: 5′-BIOTIN-CACCCTTGGTTTTTCTCAACTC-3′ (Biomers), which yield a 455-bp nucleotide sequence. PCR was performed in 25-*μ*l reactions using 25 ng DNA, 0.2 *μ*M of each primer, 0.2 mM of each deoxynucleotide (Amersham Biosciences), 1.5 mM MgCl_2_ (Applied Biosystems), 1 × PCR Gold Buffer (Applied Biosystems), and 0.5 U of AmpliTaq Gold (Applied Biosystems).

CYP2C9*3 (rs1057910) was amplified using PCR primers Fw: 5′-BIOTIN-TGCACGAGGTCCAGAGAT-3′ and Re: 5′-NNGATACTATGAATTTGGGACTTC-3′, which yield a 155-bp nucleotide sequence. PCR was performed in 25-*μ*l reactions using 25 ng DNA, 0.2 *μ*M of each primer, 0.2 mM of each deoxynucleotide (Amersham Biosciences), 1.5 mM MgCl_2_ (Applied Biosystems), 1 × PCR Gold Buffer (Applied Biosystems), and 0.5 U of AmpliTaq Gold (Applied Biosystems).

### PSQ and sequencing

The PCR products of CYP2C8*3 (G416A and A1196G), CYP2C8*4, CYP2C9*2, and CYP2C9*3 were sequenced (PyroGold, Pyrosequencing; Biotage, Uppsala, Sweden) according to the manufacturer's instructions and run on pyrosequencing (PSQ) HS 96A. The following PSQ primers were used: CYP2C8*3(G416A)_PSQ, 5′-CGGTCCTCAATGCTC-3′; CYP2C8*3(A1196G)_PSQ, 5′-ATTTGGATTAGGAAATTCT-3′; CYP2C8*4_PSQ, 5′-CAATCCTCGGGACTTT-3′; CYP2C9*2_PSQ, 5′-GGGAAGAGGAGCATTGAGGAC-3′; and CYP2C9*3_PSQ, 5′-TGGTGGGGAGAAGGTC-3′ (Biomers). Results were analysed using the inbuilt software programme on PSQ HS 96A. For quality control, every fourth sample was run in duplicate in separate PCR and PSQ reactions.

All different genotypes found in the PSQ reaction were confirmed by sequencing (Big Dye, Terminator Cycle Sequencing; Applied Biosystems) according to the manufacturer's instructions and run on an ABI 3100 Genetic Analyzer (Applied Biosystems). In April 2006, the system was upgraded to an ABI Prism 3130*xl* Genetic Analyzer (Applied Biosystems). Results were analysed using the Sequencing Analysis software (Applied Biosystems) and evaluated using the Sequencher software (Gene Codes Corporation) current version 4.5.

#### Validation

Validation was performed with separate PCR and sequencing reactions.

CYP2C8*3: Four hundred and twenty-two samples have been validated and the concordance rate was 100%.

CYP2C8*4: One hundred and ninety-two samples have been validated and the concordance rate was 100%.

CYP2C9*2: One hundred and eighty-one samples have been validated and the concordance rate was 97.2%.

A homozygote CYP2C9*2 reference sample was sometimes analysed with the PSQ software as a heterozygote instead of a homozygote. We, therefore, re-evaluated all the heterozygotes to make sure they were not in fact homozygotes, and they were not.

CYP2C9*3: One hundred and eighty-two samples have been validated and the concordance rate was 100%.

### Statistical analyses

The statistical software PASW 17.0 was used. Tumour characteristics were compared between different genotypes using Student's *t*-test for continuous variables (age and tumour size) and *χ*^2^-test for categorical variables. Mann–Whitney *U*-test was used for tumour size since this variable was not normally distributed. Tumour size was transformed using natural logarithm to obtain a better distribution for use in the multivariate linear regression models. Breast cancer-free survival rates in relation to different genotypes were assessed using Kaplan–Meier log-rank test and Cox regression models. A *P*-value less than 0.05 was considered significant. All *P*-values were two-sided. Nominal *P*-values are presented without adjustment for multiple testing.

## Results

The characteristics of the women are presented in Table 1. Of the patients, 517 were included in Lund and 135 in Helsingborg. Age at diagnosis and all tumour characteristics were comparable between patients from the two hospitals, except for histological grade, which was higher in Helsingborg patients (*χ*^2^-test *P*<0.001).

The frequencies of *CYP2C8* and *CYP2C9* polymorphisms are presented in Table 2a–c. CYP2C8*3 and CYP2C8*4 were in linkage disequilibrium (LD) and appeared not to be present on the same allele (Table 2a). Similarly, CYP2C9*2 and CYP2C9*3 were in LD and appeared to be mutually exclusive (Table 2b). As previously reported, CYP2C8*3 and CYP2C9*2 were highly, but not perfectly, linked (Table 2c). None of the polymorphisms differed significantly in frequency between patients from the two hospitals.

Based on the above information we constructed the most likely haplotypes of *CYP2C8/9* (Table 3). Six haplotypes were found in this patient population. More than half of the patients (55.5%) carried two copies of alleles with wild-type SNPs in all four positions. Another 37.7% carried one such haplotype. The second most common haplotype was the combination of CYP2C8*3 and CYP2C9*2, and was present in 17.9% of patients. CYP2C8*4 appeared only on a haplotype, with wild type SNPs in the remaining three positions, and was present in nearly 13% of the patients. CYP2C9*3 in combination with wild-type SNPs in the remaining three positions was present in over 12% of the patients. Other combinations were rare or did not exist.

### Tumour characteristics in relation to polymorphisms in CYP2C8 and CYP2C9

Patients without preoperative treatment and at least one copy of CYP2C8*3, CYP2C9*2, or CYP2C9*3 did not significantly differ with respect to age at diagnosis, invasive tumour size, axillary node status, histological grade, and ER or PR status when compared with the wild-type for each SNP. However, carrying at least one copy of CYP2C8*4 was associated with a significantly lower frequency of axillary lymph node involvement (21% *versus* 38%; *P*=0.002) in spite of a lower frequency of PR-positive tumours (57% *versus* 71%; *P*=0.012) and similar tumour sizes compared with patients without this SNP (Table 4).

### Haplotypes of CYP2C8/9 in relation to tumour characteristics

Only the haplotype allele containing the CYP2C8*4 was associated with the tumour characteristics as presented above.

### Factors associated with axillary lymph node status

Since women with the CYP2C8*4 appeared to have lower risk for lymph node involvement, we performed multivariate logistic regression analyses to elucidate which factors were associated with axillary lymph node status. Patients with invasive tumours, who had not received neo-adjuvant treatment or interstitial laser thermotherapy, were included in the analyses.

Only increasing tumour size was associated with axillary lymph node spread (*P*<0.0001), adjusted for age at diagnosis, histological grade, and ER and PR status. We then added the haplotype with the CYP2C8*4 allele to the model. The number of CYP2C8/9 *1/*4/*1/*1 copies was significantly associated with a decreased frequency for axillary lymph node involvement (odds ratio (OR) 0.40; 95% Confidence interval (CI) 0.22–0.74; *P*=0.003), adjusted for tumour size, histological grade, age at diagnosis, and ER and PR status.

In women with invasive tumour sizes of 20 mm or less, the CYP2C8/9 *1/*4/*1/*1 haplotype allele was not associated with lymph node status. Conversely, in women with tumours that were 21 mm or larger, each CYP2C8/9 *1/*4/*1/*1 allele was associated with significantly lower risk for lymph node involvement (OR 0.18, 95% CI 0.06–0.54; *P*=0.002), while each copy of the CYP2C8/9 *3/*1/*2/*1 allele was associated with a more than doubled risk of lymph node involvement (OR 2.53, 95% CI 0.97–6.64; *P*=0.06), adjusted for tumour size, histological grade, age at diagnosis, and ER and PR status. The associations between the CYP2C8/9 *1/*4/*1/*1 haplotype and CYP2C8/9 *3/*1/*2/*1 haplotypes and lymph node involvement was different depending on the invasive tumour size (exp(*β*)=0.33; *P*_*i*nteraction_=0.10 and exp(*β*)=3.2; *P*_*i*nteraction_=0.040, respectively, when entered into the same model). The effects were somewhat stronger when the interaction terms were entered individually (exp(*β*)=0.33; *P*_*i*nteraction_=0.066 for CYP2C8/9 *1/*4/*1/*1 and exp(*β*)=3.4; *P*_*i*nteraction_=0.028 for CYP2C8/9 *3/*1/*2/*1). Since CYP2C8*3 and CYP2C9*2 are not in perfect LD, we also examined each SNP separately in women with tumours 21 mm or larger. For each copy of the CYP2C8*3 allele, the adjusted odds for nodal involvement was 2.74 (95%CI 1.16–6.51; *P*=0.022). For each copy of CYP2C9*2, the adjusted OR was 2.81 (95%CI 1.18–6.67; *P*=0.020).

### Breast cancer-related events

We then investigated the association between *CYP2C8/9* haplotypes in relation to early breast cancer events (local, regional, new breast cancer, distant metastases, or death from breast cancer) by December 2008. The median follow-up time is now 25 months (range 0–62 months). During this period, 52 breast cancer-related events were reported in women with primary invasive breast cancer, excluding four women who were found to have distant metastases on the postoperative metastatic screen. Seven of these women had received some form of neoadjuvant therapy, three women had received preoperative interstitial laser thermotherapy, 14 women had received postoperative polychemotherapy, 23 women had received tamoxifen, and eight women had received an aromatase inhibitor prior to recurrence (some women received more than one adjuvant treatment). The CYP2C8 enzyme is involved in paclitaxel metabolism, but only one woman with a breast cancer-related event had been treated with a paclitaxel-based regimen and four other women with events had received a docetaxel-based regimen as neoadjuvant treatment.

The increasing number of CYP2C8*3/*1/*2/*1 alleles was associated with shorter disease-free survival in 297 ER-positive patients with invasive tumours who had received tamoxifen prior to the last follow-up or event (log rank 6.36; 1 df; *P*=0.012), HR 2.54 (95% CI 1.11–5.79; *P*=0.027), adjusted for age, tumour size, axillary lymph node status, PR status, histological grade, and other haplotypes. None of the other factors adjusted for in the model were significantly associated with early events.

**Figure 1:**
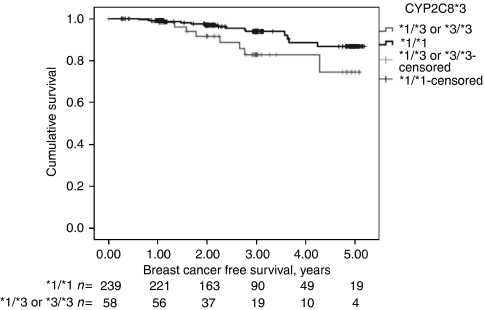
The figure shows that having at least one copy of the CYP2C8*3 allele conferred an increased risk for an early breast cancer-related event in tamoxifen-treated women with invasive ER-positive cancers (log rank 4.47; 1 df; *P*=0.034). HR 9.10 (95% CI 1.39-59.6; *P*=0.021), adjusted for invasive tumour size, age, PR, histological grade, axillary lymph node status, CYP2C8*4, CYP2C9*2, and CYP2C9*3. The number of patients at each time point is indicated.

Since there was a high degree of LD between CYP2C8*3 and CYP2C9*2, we also examined the risk of event in women the CYP2C8*3 allele. The results were stronger (log rank 8.16; 1 df; *P*=0.004), adjusted HR 8.56 (95% CI 1.53–51.1; *P*=0.015) per copy. Since there were only few women who were homozygous for CYP2C8*3, we also examined the HR for women with any CYP2C8*3 allele (log rank 4.47 1 df; *P*=0.034), adjusted HR 9.10 (95%CI 1.39–59.6; *P*=0.021) (Figure 1). There was no significant effect of carrying at least one CYP2C9*2, but it went in the opposite direction, adjusted HR 0.22 (95%CI 0.33–1.51; *P*=0.12).

## Discussion

This study is, to our knowledge, the first to have constructed *CYP2C8/9* haplotypes including the CYP2C8*4 polymorphism and to have shown that this polymorphism is unlikely to be present on the same allele as CYP2C8*3 in a Swedish population. A recent paper on a small Spanish population reported a similar finding (Dorado *et al*, 2008), but the frequency of CYP2C8*4 was much lower in that study. The frequencies of CYP2C8*3 and CYP2C8*4 were similar to those reported in the HapMap project for European populations (NCBI SNP homepage, 2008), and the frequencies of the CYP2C9*2 and CYP2C9*3 were in line with those reported by Yasar *et al* (1999) from a Swedish population of 430 unrelated healthy people.

This is the first study to report an association between decreased odds for lymph node involvement and CYP2C8*4 and an over twofold increased odds for lymph node involvement among CYP2C8/9 *3/*1/*2/*1 carriers with invasive tumour sizes over 20 mm. CYP2C8*3 and CYP2C8*4 have both been reported to have lower metabolic activity than the wild type (Bahadur *et al*, 2002), but the associations between these polymorphisms and axillary nodal involvement went in opposite directions in the current study. Since CYP2C8*3 was in strong LD with CYP2C9*2, it is likely that the association between CYP2C8*3 and nodal status is actually reflecting the association between the CYP2C9*2 polymorphism and nodal status.

We found that an increasing number of CYP2C8/9 *3/*1/*2/*1 haplotype alleles and especially CYP2C8^*^3 alleles was associated with increased hazard of early breast cancer-related events in tamoxifen-treated patients. Tamoxifen is a moderate CYP2C8 inhibitor (Walsky *et al*, 2005), a CYP2C9 substrate (Jin *et al*, 2005), and significantly inhibits CYP2C9 activity in breast cancer patients (Boruban *et al*, 2006). CYP2C9 is involved in tamoxifen activation, although neither CYP2C9*2 nor CYP2C9*3, which have lower activity than the wild type, were significantly associated with the levels of the potent tamoxifen metabolite endoxifen in one study (Jin *et al*, 2005). The effect of *CYP2C8* polymorphisms on endoxifen levels has, to our knowledge, not been investigated. Since the LD between CYP2C8*3 and CYP2C9*2 was incomplete, we also examined the effect of each SNP separately in the model, and while CYP2C8*3 was associated with significant increased hazard of early breast cancer-related events, CYP2C9*2 was associated with non-significant decreased hazard. If our finding is replicated, the CYP2C8*3 may be used to identify patients who may recur early when treated with tamoxifen and who should be offered additional or different treatment.

Currently, no genotype data are used prior to selection of tamoxifen or aromatase inhibitors for breast cancer patients with ER-positive tumours warranting adjuvant endocrine treatment. *CYP2D6* has been shown to significantly affect the levels of endoxifen and clinical outcome after tamoxifen treatment (Jin *et al*, 2005; Goetz *et al*, 2008). To our knowledge there is no association between *CYP2D6* and *CYP2C8/9*, and these genes are located on different chromosomes. It is, therefore, unlikely that the increased risk for early recurrences observed in CYP2C8*3 carriers would be explained by *CYP2D6* polymorphisms. Schroth *et al*. studied *CYP2D6*, *CYP2C19*, *CYP3A5*, *CYP2B6*, and *CYP2C9* in tamoxifen-treated women. They reported that the CYP2C19*17 allele partly compensates for non-functioning *CYP2D6* alleles and showed that carriers of one *CYP2D6*-null allele could be further stratified according to their CYP2C19*17 genotype with respect to tamoxifen response (Schroth *et al*, 2007). The genotypes of *CYP3A5*, *CYP2B6*, and *CYP2C9* were not significantly associated with tamoxifen response, which is in line with our finding with respect to CYP2C9*2 and CYP2C9*3, but they did not examine *CYP2C8*.

The St Gallen guidelines recommend that extensive peritumoral vascular invasion be used as a prognostic factor (Goldhirsch *etal*, 2007). The degree of angiogenesis is not yet routinely evaluated in the clinical setting in Sweden, and we could, therefore, not evaluate whether there was any association between peritumoral vascular invasion and *CYP2C8/9* haplotypes. It is plausible that we could have found an association, since CYP2C9-derived EETs stimulate angiogenesis (Michaelis *et al*, 2003). CYP2C9-derived EETs do so by a mechanism involving activation of the EGFR (Michaelis *et al*, 2003). One study reported high expression of EGFR to be inversely correlated with nodal metastases and shorter, distant disease-free survival in a group of breast cancer patients who had received 2 years of adjuvant tamoxifen treatment (Dihge *et al*, 2008). This is not fully compatible with our finding, as we found both a higher frequency of nodal involvement with the CYP2C8/9 *3/*1/*2/*1 genotype (in tumours larger than 20 mm) and a higher risk of any type of breast cancer recurrence, especially in women treated with tamoxifen. In small tumours, angiogenesis is also a predictor of nodal status (Arisio *et al*, 2000), and it is thus possible that women with the CYP2C8/9 *3/*1/*2/*1 genotype have tumours that are more likely to metastasise through increased angiogenesis.

The enzyme activity of CYP2C8 and CYP2C9 is not only determined by polymorphic variants, but also by use of CYP2C8- and CYP2C9-inhibiting compounds, including several drugs, as reviewed by Ingelman-Sundberg *et al* (2007). In the present study we enquired only about the use of all concomitant medications during the past week and lack information on long-term use of any of the medications. However, this information is not relevant in relation to tumour characteristics as the tumours were formed many years prior to diagnosis.

HER-2/neu status was not analysed routinely before November 2005, and we have therefore not been able to evaluate the correlation between HER-2 overamplification and *CYP2C8/9* haplotypes. Histological grade was assessed according to the procedure of Elston and Ellis (1991). In the current study histological grade was not associated with early recurrences in either group (data not shown), but the median follow-up time is still short.

Our material consisted of a series of primary breast cancer patients, where the only exclusion criteria were any previous breast cancer diagnosis and other cancer diagnosed within the past 10 years. Approximately 60% of the patients operated on in Lund and 46% of the patients operated on in Helsingborg during the study's enrolment period were included. Our sample from Lund was similar to all patients from Lund with respect to age and ER and PR status. The patients from Helsingborg were somewhat older and had fewer PR-positive tumours as compared with the region as a whole, while the subset of patients who were included in this study was comparable to those included from Lund and the whole South Swedish region with respect to age and hormone-receptor status. There are several potential explanations for why receptor status may be different in Helsingborg patients. The patients in Helsingborg are somewhat older and different sets of antibodies are used at different Departments of pathology. ER and PR status was independently re-assessed by 22 pathologists from nine hospitals and the kappa values were 0.78 for ER and 0.72 for PR in 2003 (Chebil *et al*, 2003). Provided that these markers are used for selection of breast cancer treatment, quality assurance is ongoing. The frequency of HRT use may also differ between the two cities and HRT treatment is associated with a higher frequency of ER-positive tumours (Glass *et al*, 2007). As patients who were included did not differ with respect to ER, PR, or prior HRT use, we were unable to explain why patients from Helsingborg in general would have fewer PR-positive tumours.

In conclusion, we found that the frequencies of CYP2C8*3 and CYP2C8^*^4, and CYP2C9*2 and CYP2C9^*^3 were comparable to those of healthy European populations. Each copy of the CYP2C8/9 *1/*4/*1/*1 allele was associated with significantly lower risk for nodal involvement, while each copy of the CYP2C8/9 *3/*1/*2/*1 allele was associated with increased risk for nodal involvement in tumours larger than 20 mm. Moreover, the CYP2C8*3 allele was associated with early breast cancer-related events in women treated with tamoxifen. Since this is the first study reporting an association between *CYP2C8* and tamoxifen response, and the median follow-up time is still short, the finding warrants confirmation. If confirmed, it is possible that CYP2C8*3 can be used as a genetic marker for prediction of treatment response to tamoxifen.

## Figures and Tables

**Table 1:** Preoperative characteristics of the 652 patients

	**All patients**		
	**Median or %**	**IQR**	**Missing**
Age at diagnosis (years)	59.6	50.7–67.0	0
Height (cm)	165	162–170	1
Weight (kg)	68	61–76	1
Body mass index (BMI) (kg m^−2^)	24.6	22.3–27.7	2
Waist-to-hip ratio (WHR)	0.83	0.78–0.88	4
Total breast volume (cm^3^)	1000	700–1600	9
Age at menarche (years)	13	12–14	5
Premenopausal	24.5%		4
Age at menopause	50	47–52	192
Age at first full-term pregnancy^a^	24	21–28	96
Parity	2	1–3	0
Ever oral contraceptive use	70.1%		0
Ever hormone replacement therapy use	45.6%		1
Abstainer of alcohol (%)	12.1%		1
Current smoker (%)	22.0%		1
Daily coffee consumption (cups)	3	2–4	2
1° relative with breast cancer	19.2%		21

Abbreviation: IQR=interquartile range.

a Among parous women only.

**Table 2:** (a–c) The relationship between *CYP2C8* and *CYP2C9* genotypes in the breast cancer patient group

*(a)*	CYP2C8*1/*1	CYP2C8*1/*4	CYP2C8*4/*4	*n*
CYP2C8*1/*1	445	76	3	524
CYP2C8*1/*3	116	5	0	121
CYP2C8*3/*3	7	0	0	7
*n*	568	81	3	652
				
*(b)*	CYP2C9*1/*1	CYP2C9*1/*3	CYP2C9*3/*3	*n*
CYP2C9*1/*1	438	67	2	507
CYP2C9*1/*2	124	11	0	135
CYP2C9*2/*2	8	0	0	8
*n*	570	78	2	650
				
*(c)*	CYP2C9*1/*1	CYP2C9*1/*2	CYP2C9*2/*2	*n*
CYP2C8*1/*1	498	25	1	524
CYP2C8*1/*3	9	110	1	120
CYP2C8*3/*3	0	1	6	7
*n*	507	136	8	652

Abbreviation: CYP=cytochrome P450.

Table 2a shows linkage disequilibrium (LD) between the CYP2C8*3 and CYP2C8*4 genotypes among the 652 breast cancer patients included. Table 2b shows LD between the CYP2C9*2 and CYP2C9*3 genotypes. Table 2c confirms the previously reported imperfect LD between CYP2C8*3 and CYP2C9*2. Genotype data on CYP2C9*2 were missing for one woman and CYP2C9*3 genotype data were missing for another woman.

**Table 3:** shows the frequencies of the most likely *CYP2C8/9* haplotypes sorted according to their frequency among the breast cancer patients

	**No allele**	**One allele**	**Two alleles**	**Missing**
CYP2C8/9^*^1/^*^1/^*^1/^*^1	42 (6.4)	246 (37.7)	362 (55.5)	2 (0.3)
CYP2C8/9^*^3/^*^1/^*^2/^*^1	533 (81.7)	111 (17.0)	6 (0.9)	2 (0.3)
CYP2C8/9^*^1/^*^4/^*^1/^*^1	566 (86.8)	81 (12.4)	3 (0.5)	2 (0.3)
CYP2C8/9^*^1/^*^1/^*^1/^*^3	570 (87.4)	78 (12.0)	2 (0.3)	2 (0.3)
CYP2C8/9^*^1/^*^1/^*^2/^*^1	623 (95.6)	26 (4.0)	1 (0.2)	2 (0.3)
CYP2C8/9^*^3/^*^1/^*^1/^*^1	640 (98.2)	10 (1.5)	0 (0.0)	2 (0.3)

Abbreviation: CYP=cytochrome P450.

These were constructed based on the information obtained from Tables 2a–c.

**Table 4:** Characteristics of the 612 tumours from the patients not treated with preoperative interstitial laser thermo therapy (*n*=11 and 1 uncertain) or neoadjuvant therapy (*n*=28) in this study

	***n* (%)**	Any CYP2C8*4 allele (%)
*Invasive tumour size*		
p_is_	14 (2.3)	28.6
pT1	432 (70.6)	12.0
PT2	152 (24.8)	13.8
pT3	12 (12.0)	8.3
pT4	1 (0.2)	0
Missing	3 (0.5)	
		
*Axillary lymph node involvement*		
pN−	389 (63.8)	15.9
pN+	220 (35.9)	7.3
Missing	3 (0.5)	
		
*Histological grade*		
Grade 1	156 (25.5)	9.3
Grade 2	312 (51.0)	12.2
Grade 3	142 (23.2)	16.9
Missing	3 (0.5)	
		
*Hormone receptor status*		
ER+/PR+	411 (67.2)	10.2
ER+/PR−	111 (18.1)	16.2
ER−/PR−	72 (11.8)	19.4
ER−/PR+	4 (0.7)	0
Missing	13 (2.1)	

Abbreviations: CYP=cytochrome P450; ER=oestrogen receptor; PR=progesterone receptor.

The CYP2C8*4 allele was the only genotype that was associated with tumour characteristics. The percentage of patients with at least one CYP2C8*4 allele is indicated in relation to the tumour characteristics.

^*^Large and inflammatory tumours were treated by neoadjuvant therapy; therefore there is no information presented on these tumours.

## References

[bib1] Arisio R, Sapino A, Cassoni P, Accinelli G, Cuccorese MC, Mano MP, Bussolati G (2000) What modifies the relation between tumour size and lymph node metastases in T1 breast carcinomas? J Clin Pathol 53: 846–850 11127267 10.1136/jcp.53.11.846 PMC1731116

[bib2] Bahadur N, Leathart JB, Mutch E, Steimel-Crespi D, Dunn SA, Gilissen R, Houdt JV, Hendrickx J, Mannens G, Bohets H, Williams FM, Armstrong M, Crespi CL, Daly AK (2002) CYP2C8 polymorphisms in Caucasians and their relationship with paclitaxel 6alpha-hydroxylase activity in human liver microsomes. Biochem Pharmacol 64: 1579–1589 12429347 10.1016/s0006-2952(02)01354-0

[bib3] Belton O, Fitzgerald DJ (2003) Cyclooxygenase isoforms and atherosclerosis. Expert Rev Mol Med 5: 1–18 10.1017/S1462399403005842 14987412

[bib4] Boruban MC, Yasar U, Babaoglu MO, Sencan O, Bozkurt A (2006) Tamoxifen inhibits cytochrome P450 2C9 activity in breast cancer patients. J Chemother 18: 421–424 17024799 10.1179/joc.2006.18.4.421

[bib5] Chebil G, Bendahl PO, Ferno M (2003) Estrogen and progesterone receptor assay in paraffin-embedded breast cancer—reproducibility of assessment. Acta Oncol 42: 43–47 12665330 10.1080/02841860300672

[bib6] Dai D, Zeldin DC, Blaisdell JA, Chanas B, Coulter SJ, Ghanayem BI, Goldstein JA (2001) Polymorphisms in human CYP2C8 decrease metabolism of the anticancer drug paclitaxel and arachidonic acid. Pharmacogenetics 11: 597–607 11668219 10.1097/00008571-200110000-00006

[bib7] Dihge L, Bendahl PO, Grabau D, Isola J, Lovgren K, Ryden L, Ferno M (2008) Epidermal growth factor receptor (EGFR) and the estrogen receptor modulator amplified in breast cancer (AIB1) for predicting clinical outcome after adjuvant tamoxifen in breast cancer. Breast Cancer Res Treat 109: 255–262 17636398 10.1007/s10549-007-9645-1

[bib8] Dorado P, Cavaco I, Caceres MC, Piedade R, Ribeiro V, Llerena A (2008) Relationship between CYP2C8 genotypes and diclofenac 5-hydroxylation in healthy Spanish volunteers. Eur J Clin Pharmacol 64: 967–970 18548238 10.1007/s00228-008-0508-4

[bib9] Early breast cancer trialists' collaborative group (2005) Effects of chemotherapy and hormonal therapy for early breast cancer on recurrence and 15-year survival: an overview of the randomised trials. Lancet 365: 1687–1717 15894097 10.1016/S0140-6736(05)66544-0

[bib10] Elston CW, Ellis IO (1991) Pathological prognostic factors in breast cancer. I. The value of histological grade in breast cancer: experience from a large study with long-term follow-up. Histopathology 19: 403–410 1757079 10.1111/j.1365-2559.1991.tb00229.x

[bib11] Forbes JF, Cuzick J, Buzdar A, Howell A, Tobias JS, Baum M (2008) Effect of anastrozole and tamoxifen as adjuvant treatment for early-stage breast cancer: 100-month analysis of the ATAC trial. Lancet Oncol 9: 45–53 18083636 10.1016/S1470-2045(07)70385-6

[bib12] Glass AG, Lacey Jr JV, Carreon JD, Hoover RN (2007) Breast cancer incidence, 1980–2006: combined roles of menopausal hormone therapy, screening mammography, and estrogen receptor status. J Natl Cancer Inst 99: 1152–1161 17652280 10.1093/jnci/djm059

[bib13] Goetz MP, Kamal A, Ames MM (2008) Tamoxifen pharmacogenomics: the role of CYP2D6 as a predictor of drug response. Clin Pharmacol Ther 83: 160–166 17882159 10.1038/sj.clpt.6100367 PMC2752373

[bib14] Goldhirsch A, Wood WC, Gelber RD, Coates AS, Thurlimann B, Senn HJ (2007) Progress and promise: highlights of the international expert consensus on the primary therapy of early breast cancer 2007. Ann Oncol 18: 1133–1144 17675394 10.1093/annonc/mdm271

[bib15] Griskevicius L, Yasar U, Sandberg M, Hidestrand M, Eliasson E, Tybring G, Hassan M, Dahl ML (2003) Bioactivation of cyclophosphamide: the role of polymorphic CYP2C enzymes. Eur J Clin Pharmacol 59: 103–109 12684728 10.1007/s00228-003-0590-6

[bib16] Ingelman-Sundberg M, Sim SC, Gomez A, Rodriguez-Antona C (2007) Influence of cytochrome P450 polymorphisms on drug therapies: pharmacogenetic, pharmacoepigenetic and clinical aspects. Pharmacol Ther 116: 496–526 18001838 10.1016/j.pharmthera.2007.09.004

[bib17] Jiang JG, Ning YG, Chen C, Ma D, Liu ZJ, Yang S, Zhou J, Xiao X, Zhang XA, Edin ML, Card JW, Wang J, Zeldin DC, Wang DW (2007) Cytochrome P450 epoxygenase promotes human cancer metastasis. Cancer Res 67: 6665–6674 17638876 10.1158/0008-5472.CAN-06-3643

[bib18] Jin Y, Desta Z, Stearns V, Ward B, Ho H, Lee KH, Skaar T, Storniolo AM, Li L, Araba A, Blanchard R, Nguyen A, Ullmer L, Hayden J, Lemler S, Weinshilboum RM, Rae JM, Hayes DF, Flockhart DA (2005) CYP2D6 genotype, antidepressant use, and tamoxifen metabolism during adjuvant breast cancer treatment. J Natl Cancer Inst 97: 30–39 15632378 10.1093/jnci/dji005

[bib19] King BP, Khan TI, Aithal GP, Kamali F, Daly AK (2004) Upstream and coding region CYP2C9 polymorphisms: correlation with warfarin dose and metabolism. Pharmacogenetics 14: 813–822 15608560 10.1097/00008571-200412000-00004

[bib20] Malmström P, Bendahl PO, Boiesen P, Brünner N, Idvall I, Fernö M, South Sweden Breast Cancer Group (2001) S-phase fraction and urokinase plasminogen activator are better markers for distant recurrences than Nottingham Prognostic Index and histologic grade in a prospective study of premenopausal lymph node-negative breast cancer. J Clin Oncol 19: 2010–2019 11283134 10.1200/JCO.2001.19.7.2010

[bib21] Malmström P, Holmberg L, Anderson H, Mattsson J, Jönsson PE, Tennvall-Nittby L, Balldin G, Loven L, Svensson JH, Ingvar C, Möller T, Holmberg E, Wallgren A, Swedish Breast Cancer Group (2003) Breast conservation surgery, with and without radiotherapy, in women with lymph node-negative breast cancer: a randomised clinical trial in a population with access to public mammography screening. Eur J Cancer 39: 1690–1697 12888363 10.1016/s0959-8049(03)00324-1

[bib22] Medhora M, Daniels J, Mundey K, Fisslthaler B, Busse R, Jacobs ER, Harder DR (2003) Epoxygenase-driven angiogenesis in human lung microvascular endothelial cells. Am J Physiol Heart Circ Physiol 284: H215–H224 12388259 10.1152/ajpheart.01118.2001

[bib23] Michaelis UR, Fisslthaler B, Barbosa-Sicard E, Falck JR, Fleming I, Busse R (2005) Cytochrome P450 epoxygenases 2C8 and 2C9 are implicated in hypoxia-induced endothelial cell migration and angiogenesis. J Cell Sci 118: 5489–5498 16291720 10.1242/jcs.02674

[bib24] Michaelis UR, Fisslthaler B, Medhora M, Harder D, Fleming I, Busse R (2003) Cytochrome P450 2C9-derived epoxyeicosatrienoic acids induce angiogenesis via cross-talk with the epidermal growth factor receptor (EGFR). FASEB J 17: 770–772 12586744 10.1096/fj.02-0640fje

[bib25] NCBI SNP homepage (2008) NCBI SNP homepage Access date 2008-07-15 http://www.ncbi.nlm.nih.gov/SNP/snp_ref.cgi?rs=1058930

[bib26] Sandberg M, Johansson I, Christensen M, Rane A, Eliasson E (2004) The impact of CYP2C9 genetics and oral contraceptives on cytochrome P450 2C9 phenotype. Drug Metab Dispos 32: 484–489 15100169 10.1124/dmd.32.5.484

[bib27] Schroth W, Antoniadou L, Fritz P, Schwab M, Muerdter T, Zanger UM, Simon W, Eichelbaum M, Brauch H (2007) Breast cancer treatment outcome with adjuvant tamoxifen relative to patient CYP2D6 and CYP2C19 genotypes. J Clin Oncol 25: 5187–5193 18024866 10.1200/JCO.2007.12.2705

[bib28] Walsky RL, Gaman EA, Obach RS (2005) Examination of 209 drugs for inhibition of cytochrome P450 2C8. J Clin Pharmacol 45: 68–78 15601807 10.1177/0091270004270642

[bib29] Yasar U, Eliasson E, Dahl ML, Johansson I, Ingelman-Sundberg M, Sjoqvist F (1999) Validation of methods for CYP2C9 genotyping: frequencies of mutant alleles in a Swedish population. Biochem Biophys Res Commun 254: 628–631 9920790 10.1006/bbrc.1998.9992

[bib30] Yasar U, Lundgren S, Eliasson E, Bennet A, Wiman B, de Faire U, Rane A (2002) Linkage between the CYP2C8 and CYP2C9 genetic polymorphisms. Biochem Biophys Res Commun 299: 25–28 12435384 10.1016/s0006-291x(02)02592-5

[bib31] Zeldin DC, DuBois RN, Falck JR, Capdevila JH (1995) Molecular cloning, expression and characterization of an endogenous human cytochrome P450 arachidonic acid epoxygenase isoform. Arch Biochem Biophys 322: 76–86 7574697 10.1006/abbi.1995.1438

